# MIR22HG Aggravates Oxygen-Glucose Deprivation and Reoxygenation-Induced Cardiomyocyte Injury through the miR-9-3p/SH2B3 Axis

**DOI:** 10.1155/2022/7332298

**Published:** 2022-05-31

**Authors:** Yi Ge, Lishi Liu, Liang Luo, Yu Fang, Tong Ni

**Affiliations:** Department of Intensive Care Unit, Wuxi Second People's Hospital, Wuxi, 214000 Jiangsu, China

## Abstract

Reperfusion therapy, the standard treatment for acute myocardial infarction (MI), can trigger necrotic death of cardiomyocytes and provoke ischemia/reperfusion (I/R) injury. However, molecular mechanisms that regulate cardiomyocyte death remain largely unknown. The abnormal expression of lncRNA MIR22HG has been found in types of diseases. The current study was aimed at exploring the function and mechanism of MIR22HG in I/R injury. In this study, mouse myocardial cells (HL-1) treated with oxygen-glucose deprivation and reoxygenation (OGD/R) were used as the *in vitro* models, and myocardial ischemia reperfusion injury (MIRI) animal models *in vivo* were established in male C57BL/6 mice. Experiments including CCK-8, flow cytometry, TUNEL, HE staining, RT-qPCR, western blotting, and luciferase reporter assays were performed to explore the function and potential mechanism of MIR22HG in MIRI *in vitro* and *in vivo*. Bioinformatics analysis was performed to predict the binding site between miR-9-3p and MIR22HG (or SH2B3). Our results indicated that the MIR22HG level was upregulated in cardiomyocytes after OGD/R treatment. The knockdown of MIR22HG promoted cell viability and inhibited apoptosis and extracellular matrix (ECM) production in OGD/R-treated HL-1 cells. In mechanism, MIR22HG binds to miR-9-3p, and miR-9-3p targets the SH2B3 3′ untranslated region (UTR). Moreover, SH2B3 expression was positively regulated by MIR22HG but negatively modulated by miR-9-3p. Rescue assays suggested that the suppressive effect of MIR22HG knockdown on cell viability, apoptosis, and ECM accumulation was reversed by the overexpression of SH2B3. The *in vivo* experiments demonstrated that MIR22HG knockdown alleviated cardiomyocyte apoptosis and reduced myocardial infarct size in MIRI mice. In summary, MIR22HG knockdown alleviates myocardial injury through the miR-9-3p/SH2B3 axis.

## 1. Introduction

Acute myocardial infarction (AMI) is a major threat to human health with a high morbidity and mortality worldwide [1]. AMI is characterized by damage to myocardial tissue and cell death resulting from sudden deficiency of blood flow and therefore lack of nutrients and oxygen in a region of the heart [2, 3]. Reperfusion therapy, including thrombolysis, is the most effective treatment for AMI [4, 5]. The response to myocardial ischemia-reperfusion (I/R) may have paradoxical effects. It is necessary to restore nutrient and oxygen supply, and reperfusion can exacerbate cell damage in a process known as myocardial ischemia reperfusion injury (MIRI) [6]. The mechanisms underlying MIRI are complex and involve excessive formation of reactive oxygen species as a result of mitochondrial dysfunction, intracellular calcium overload, proteolysis, and finally dead or necrotic cardiomyocytes [7]. Emerging evidence has indicated that I/R can mediate injury and apoptosis of cardiomyocytes and induce abnormal accumulation of extracellular matrix (ECM) [8, 9], which ultimately contributes to lethal MI. Studies have found that about 50% of myocardial necrosis cases are caused by MIRI [10]. The mechanisms and signals involved in this damaging process remain largely elusive. This lack of knowledge aggravates the definition of AMI outcomes and diagnostic and prognostic factors, hindering progress in health care [11]. Therefore, it is important to find out how to attenuate MIRI and improve the efficacy of reperfusion therapy in AMI.

Noncoding RNAs (ncRNAs) are transcripts that are unable to encode protein [12]. It is generally divided into two groups: regulatory ncRNAs and housekeeping ncRNAs. Regulatory ncRNAs include long ncRNAs (lncRNAs), circular RNAs, and microRNAs (miRNAs) [13]. In the current study, we pay attention to the role of lncRNA and miRNA in MIRI. LncRNAs consist of more than 200 nucleotides in length [14]. Dysregulated lncRNAs are involved in various biological processes [15]. For example, lncRNA MALAT1 is upregulated in diabetic nephropathy and participates in the regulation of podocyte injury by interacting with *β*-catenin [16]. Overexpression of lncRNA H19 plays a protective role in LPS-induced injury by targeting the miR-181a/Runx2 axis and activating Notch and JNK signaling pathways [17]. LncRNA MIR22HG has been widely reported to be dysregulated in various diseases [18–20]. Although upregulation of MIR22HG expression has been identified in MI patients [21], the specific function and molecular mechanism of MIR22HG in MIRI have not been investigated.

MiRNAs are a group of single-chain and short-stranded RNAs with 19-24 nucleotides, which widely express in many eukaryotes and viruses [22, 23]. Functionally, despite the lack of capacity encoding protein, miRNAs can bind to the 3′ untranslated region (UTR) of target mRNAs to regulate their expression at the posttranscriptional level [22]. Dysfunction of miRNAs has been reported to be involved in multiple biological processes, such as cell proliferation, differentiation, autophagy, fibrosis, and apoptosis [24, 25]. MiR-335-5p alleviates chondrocyte inflammation by promoting autophagy in osteoarthritis [26]. The miRNA-23a/CXCR4 axis modulates neuropathic pain by affecting the TXNIP/NLRP3 inflammasome [27]. Recently, miR-9-3p has been demonstrated to be dysregulated in Parkinson's disease [28]. Additionally, miR-9-3p regulates renal tubular epithelial cells injury induced by cisplatin by targeting several mRNAs [29]. However, the role of miR-9-3p in MIRI remains unclear.

In this study, we carried out *in vitro* and *in vivo* experiments to judge the potential therapeutic use of MIR22HG using the oxygen-glucose deprivation/restoration- (OGD/R-) injured mouse myocardial cells and the MIRI-injured mice.

## 2. Materials and Methods

### 2.1. Oxygen-Glucose Deprivation and Reoxygenation (OGD/R) Treatment

HL-1 cells (Culture Collection of Chinese Academy of Science, Shanghai, China) were cultured in Dulbecco's modified Eagle medium (DMEM; Gibco, USA) containing 10% fetal bovine serum (FBS, Gibco) in a humidified atmosphere with 95% air and 5% CO_2_ at 37°C. As described previously [30], for OGD/R treatment, HL-1 cells were cultured in serum and glucose-free DMEM for 3 h at 37°C and then moved in a hypoxia chamber with 5% CO_2_ and 95% N_2_. After incubation for 6 h at 37°C, HL-1 cells were reoxygenated for 12 h in normal DMEM containing 10% FBS (4.5 mg/ml glucose) with 95% air and 5% CO_2_ at 37°C. HL-1 cells in the control group were cultured in a normal DMEM under normoxia.

### 2.2. Cell Transfection

Vector pcDNA3.1/SH2B3/MIR22HG (SH2B3/MIR22HG) for SH2B3/MIR22HG overexpression was constructed by cloning full-length SH2B3/MIR22HG into the pcDNA3.1 vector, and the empty pcDNA3.1 acted as a control. Short hairpin RNAs (shRNAs) targeting MIR22HG or SH2B3 (shMIR22HG or sh-SH2B3) were used to knock down MIR22HG or SH2B3 and sh-NC acted as a negative control. MiR-9-3p mimics (miR-9-3p) were used to overexpress miR-9-3p and NC mimics were used as negative controls. The transfection was performed using Lipofectamine 2000 reagent (Invitrogen, CA, USA) following the manufacturer's instructions. All plasmids were synthesized by GenePharma (Shanghai, China). After transfection for 48 h, the cells were subjected to hypoxia for 6 h and reoxygenation for 12 h, and the resultant cells were collected for subsequent experiments.

### 2.3. Real-Time Reverse-Transcription Polymerase Chain Reaction (RT-qPCR)

RNA samples from myocardial tissues or cells were extracted using TRIzol reagent (Invitrogen). PrimeScript RT reagent Kit (Takara Biotechnology Ltd., Dalian, China) was used to reverse transcribed RNA into cDNA. Subsequently, the expression of MIR22HG, miRNAs, and mRNA was examined by using SYBR Green PCR Master Mix Kit (TOYOBO, Osaka, Japan) based on an ABI 7500 Fast Real-Time PCR system (Applied Biosystems, Foster City, USA). U6 and GAPDH acted as internal references. Relative expression was determined using the 2^−*ΔΔ*CT^ method. This assay was performed for 3 times (*n* = 3).

### 2.4. Western Blotting

Total protein from myocardial tissues or cells was extracted using Protein Lysis Buffer (Beyotime, Shanghai, China). The protein concentration was determined using BCA Protein Assay Kit (Beyotime). Equal amounts of protein were separated by 10% sodium dodecyl sulphate-polyacrylamide gel electrophoresis (SDS-PAGE) and moved onto polyvinylidene fluoride (PVDF) membranes. Next, the membranes were blocked with defatted milk and incubated with primary antibodies overnight at 4°C. After washing with PBS, the membranes were incubated with secondary antibodies goat antirabbit IgG (1 : 10,000, ab6721) for 2 h at room temperature. Finally, the protein bands were visualized with enhanced chemiluminescence detection kits (Amersham Pharmacia Biotech, UK) and analyzed by ImageJ software. The primary antibodies against Bax (1 : 1000, ab32503), collagen I (1 : 1000, ab34710), SH2B3 (1 : 1000, ab191904), collagen III (1 : 1000, ab7778), Bcl-2 (1 : 1000, ab182858), fibronectin (1 : 1000, ab2413), and GAPDH (ab245356) were purchased from Abcam (Cambridge, USA) and GAPDH acted as a loading control. This assay was performed for 3 times (*n* = 3).

### 2.5. CCK-8 Assay

The viability of HL-1 cardiomyocytes was examined using Cell Counting Kit-8 (CCK-8; Dojindo, Kumamoto, Japan). After transfection and OGD/R treatment, HL-1 cells (10,000/well) were seeded in 96-well plates and 10 *μ*l of CCK-8 solution was added to each well for another incubation for 1 h at 37°C. Finally, the cell viability was assessed with a microplate reader (Bio-Rad, Shanghai, China) by measuring absorbance at 450 nm. This assay was performed for 3 times (*n* = 3).

### 2.6. Flow Cytometry Analysis

After transfection and OGD/R treatment, HL-1 cells were suspended in 195 ml of binding buffer at 5 × 10^5^ cells/ml. Next, 5 ml of Annexin V-FITC solution (Sangon Biotech, Shanghai, China) was added, and the mixture was cultured for 15 min in the dark at room temperature. After centrifugation at ×·1000 *g* for 5 min and washing with binding buffer, cells were resuspended in the mixture of 190 ml of binding buffer and 10 ml of propidium iodide (PI; Sangon Biotech). Cells were further analyzed using flow cytometry (Beckman Coulter, Fullerton, CA, USA). This assay was performed for 3 times (*n* = 3).

### 2.7. Luciferase Reporter Assay

The binding site of SH2B3 3′ UTR (or MIR22HG) in miR-9-3p is predicted in starBase database and subcloned into the pmirGLO dual-luciferase reporter (Promega, USA) to construct wild-type or mutant pmirGLO-SH2B3-3′UTR (or pmirGLO-MIR22HG) vectors. The wild-type or mutant pmirGLO-SH2B3-3′ UTR (or pmirGLO-MIR22HG) vectors were cotransfected into HL-1 cells with miR-9-3p or NC mimics using Lipofectamine 2000 (Invitrogen). Luciferase activities were measured using dual-luciferase reporter assay system (Promega) after cotransfection for 48 h. The relative luciferase activity is expressed as the ratio of firefly/renilla luciferase activity. This assay was performed for 3 times (*n* = 3).

### 2.8. Construction of a Mouse Model of MIRI

All animal experiments were performed in strict accordance with the Guide for the Care and Use of Laboratory Animals of the National Institutes of Health. Great efforts were made to minimize pain, discomfort, and suffering of all the included animals. Twenty-four male C57BL/6 mice aged 8 weeks were purchased from Beijing Vital River Laboratory Animal Technology Corporation (Beijing, China). They were housed with a 12 h of light/dark cycle and given free access to water and food. Animals were anesthetized by injecting 100 mg/kg ketamine and 10 mg/kg xylazine. After this, the left thorax of the mice was opened. Myocardial ischemia was induced by ligating the left anterior descending coronary artery (LAD) using a 6/0 round needle and a thread. Thirty minutes after the ischemia induction, the ligature was untied followed by reperfusion for 60 min. Finally, the chest cavity was sutured. The mice in the sham group were subjected to the same procedures except LAD ligation. Mice were anesthetized with isoflurane and then euthanized after experiment. The myocardial tissues were collected from infarcted area for subsequent experiments.

### 2.9. Lentiviral Injection

The lentivirus packaging sh-MIR22HG (LV-sh-MIR22HG) and negative control (sh-LV-NC) were synthesized by Hanheng Biotechnology Co., Ltd. (Shanghai, China). The anesthetized mice were hooked up to an animal ventilator, and their thorax was opened. Approximately 2 × 10^7^ pfu/ml lentivirus was injected from the apex of the left ventricle into the aortic root. Subsequently, the pulmonary artery was clamped for 20 seconds, and the heart pumped into the closed system. The chest was sutured, and the mice were sent to cages for recovery. Five days after the injection, the mice were subjected to MIRI modeling as the above mentioned, with 6 mice in each group (*n* = 6).

### 2.10. Hematoxylin-Eosin (HE) Staining

The left ventricle of heart tissues was fixed with 10% formaldehyde solution, dehydrated in ethanol gradient, embedded in paraffin, and cut into 4 *μ*m sections. After dewaxing, the samples were stained with hematoxylin and eosin. Then, the samples were fixed and observed under a light microscope (Leica Microsystems, Wetzlar, Germany). This assay was performed for 3 times (*n* = 3).

### 2.11. Terminal Deoxynucleotidyl Transferase-Mediated dUTP-Biotin Nick End Labeling (TUNEL) Staining

The heart tissues were immediately cut into 5-*μ*m-thick sections after reperfusion. Cardiomyocyte apoptosis was assessed by TUNEL staining using a DeadEnd™ Fluorometric TUNEL System as per the manufacturer's instructions (Promega Corporation, Madison, WI, USA). The sections were washed with PBS and incubated in 20 *μ*g/ml proteinase K for 10 min. After washing with PBS (0.05 M phosphate buffer containing 0.145 M sodium chloride, pH 7.4), tissue sections were incubated with equilibration buffer and then with TdT enzyme in a humidified chamber at 37°C for 1 h. Next, tissue sections were transferred into prewarmed working strength stop wash buffer for 15 min. Following rinsing with PBS, tissue sections were mounted with VECTASHIELD® antifade mounting medium containing DAPI (Vector Labs, Burlingame, California). Tissue sections on the slides were covered with a cover glass. Nuclei were stained blue with DAPI, and localized green fluorescence of apoptotic cells was detected by fluorescence microscopy and photographed (Nikon Eclipse TE2000-U, Photometrics Cool Snap cf, HCImage). TUNEL positive cells were counted manually using ImageJ (NIH) software in five randomly selected microscopic fields viewed.

### 2.12. Statistical Analysis

Data are shown as the mean ± SD and analyzed with SPSS (version 17.0, Chicago, USA). Each experiment was performed for at least three times independently to obtain mean value as well as reduce errors. Unpaired Student's *t*-test was used to compare differences between two groups and one-way ANOVA followed by Tukey's *post hoc* test was used to analyze statistical differences among multiple groups. The value of *p* < 0.05 was considered statistically significant.

## 3. Result

### 3.1. The Treatment with OGD/R Promotes Cell Apoptosis and ECM Accumulation in HL-1 Cells

**Figure 1:**
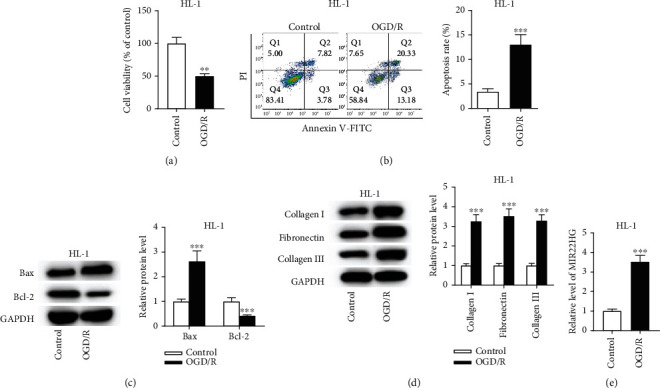
The treatment with OGD/R promotes cell apoptosis and ECM accumulation in HL-1 cells. (a) The viability of HL-1 cells treated with or without OGD/R was measured by CCK-8 assay (*n* = 3). (b) The apoptosis rate of HL-1 cells treated with or without OGD/R was determined by flow cytometry analysis (*n* = 3). (c and d) The protein levels of Bax, Bcl-2, collagen I, fibronectin, and collagen III protein levels in HL-1 cells were measured by western blotting analysis (*n* = 3). (e) The expression level of MIR22HG in OGD/R-treated HL-1 cells was measured by RT-qPCR analysis (*n* = 3). ^∗∗^*p* < 0.01,^∗∗∗^*p* < 0.001.

HL-1 cells treated with OGD/R were used as the *in vitro* models. The CCK-8 results showed that OGD/R significantly inhibited HL-1 cell viability (Figure 1(a)). The cell apoptosis was promoted by OGD/R (Figure 1(b)). Furthermore, western blotting analysis revealed that OGD/R increased Bax protein expression and reduced Bcl-2 protein expression in HL-1 cells (Figure 1(c)) as well as upregulated collagen I, fibronectin, and collagen III protein expression levels (Figure 1(d)). To explore the effect of MIR22HG in I/R injury, its expression level was determined. The RT-qPCR results showed that the MIR22HG level was increased in HL-1 cells after OGD/R treatment (Figure 1(e)), suggesting that MIR22HG may participate in the pathophysiological process of I/R injury.

### 3.2. MIR22HG Knockdown Attenuates OGD/R-Induced Cardiomyocyte Injury

**Figure 2:**
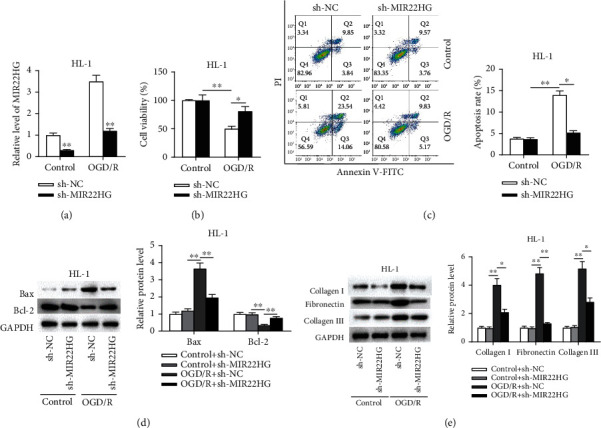
MIR22HG knockdown attenuated OGD/R-treated cardiomyocyte injury. (a) The knockdown efficiency of sh-MIR22HG was determined by RT-qPCR (*n* = 3). (b) The effect of sh-MIR22HG on the viability of OGD/R-treated HL-1 cells was assessed by CCK-8 assay (*n* = 3). (c) The effect of sh-MIR22HG on the apoptosis of OGD/R-treated HL-1 cells was evaluated by flow cytometry analysis (*n* = 3). (d and e) The effect of sh-MR22HG on the protein levels of Bax, Bcl-2, collagen I, fibronectin, and collagen III in OGD/R-treated HL-1 cells was measured by western blotting analysis (*n* = 3). ^∗^*p* < 0.05, ^∗∗^*p* < 0.01.

To investigate the function of MIR22HG in OGD/R cell models, subsequent experiments were conducted. First, the expression level of MIR22HG was successfully knocked down after transfecting sh-MIR22HG into OGD/R-treated HL-1 cells and normal cells (Figure 2(a)). The CCK-8 assay showed that the viability of OGD/R-treated cardiomyocytes was increased after silencing MIR22HG while the viability of normal cells had no change in response to MIR22HG knockdown (Figure 2(b)). Knockdown of MIR22HG significantly suppressed the apoptosis of OGD/R-treated cardiomyocytes (Figure 2(c)). Furthermore, western blotting analysis showed that silencing MIR22HG prevented the effect of ODG/R on the protein levels of Bax and Bcl-2 (Figure 2(d)), as well as the protein levels of collagen I, fibronectin, and collagen III (Figure 2(e)). Overall, silencing MIR22HG inhibits apoptosis and ECM accumulation in OGD/R-treated HL-1 cells.

### 3.3. MIR22HG Interacts with miR-9-3p to Regulate SH2B3 Expression

**Figure 3:**
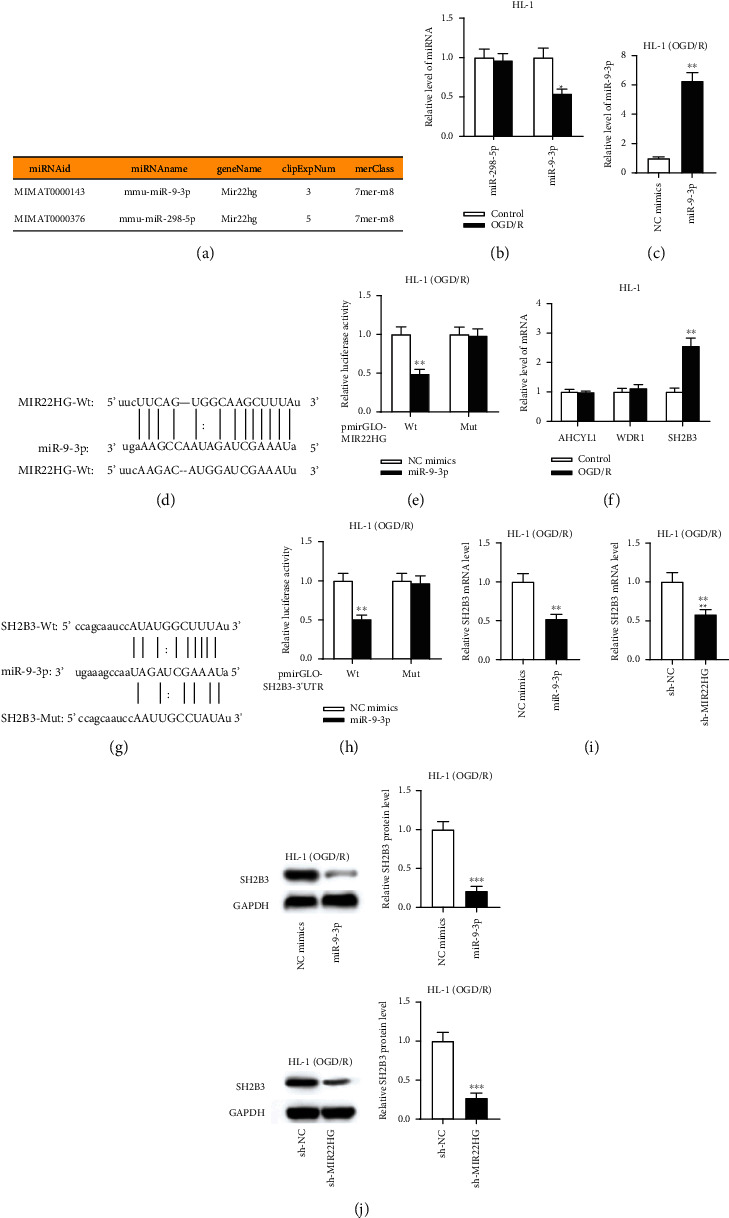
MIR22HG interacts with miR-9-3p to regulate SH2B3 expression. (a) The possible miRNAs that possibly bind to MIR22HG predicted in starBase. (b) The expression level of miR-9-3p and miR-298-5p in OGD/R-treated HL-1 cells was determined by RT-qPCR (*n* = 3). (c) The overexpression efficiency of miR-9-3p was evaluated by RT-qPCR (*n* = 3). (d and e) The binding sequence between miR-9-3p and MIR22HG is shown. Luciferase reporter assay was used to confirm the binding capacity between miR-9-3p and MIR22HG (*n* = 3). (f) The expression level of candidate target mRNAs in OGD/R-treated HL-1 cells was determined by RT-qPCR (*n* = 3). (g) The binding sequence between miR-9-3p and SH2B3 3′ UTR. (h) Luciferase reporter assay was used to validate the binding between miR-9-3p and SH2B3 3′ UTR (*n* = 3). (i and j) The mRNA and protein levels of SH2B3 in the context of miR-9-3p overexpression or MIR22HG knockdown were measured by RT-qPCR and western blotting analyses (*n* = 3). ^∗^*p* < 0.05, ^∗∗^*p* < 0.01, ^∗∗∗^*p* < 0.001.

We then investigated the molecular mechanism of MIR22HG. Several studies have verified a ceRNA network of MIR22HG in hepatocellular carcinoma and endometrial carcinoma [20, 31]. Thus, we hypothesized that MIR22HG also exerts ceRNA functions in I/R injury. The starBase website (http://starbase.sysu.edu.cn) was searched to predict the potential miRNAs containing binding site for MIR22HG, and miR-9-3p and miR-298-5p were found (search condition: medium stringency of CLIP Data) (Figure 3(a)). To identify the miRNA interacting with MIR22HG, the expression of these two miRNAs in OGD/R-treated HL-1 cells was measured. We found that miR-9-3p expression was downregulated in HL-1 cells after OGD/R treatment while miR-298-5p expression showed no significant change (Figure 3(b)). The RT-qPCR results demonstrated that the miR-9-3p level was significantly elevated by miR-9-3p mimics (Figure 3(c)). Wild-type or mutant binding sequence of MIR22HG in miR-9-3p was inserted into the pmirGLO vector (Figure 3(d)). Luciferase reporter assay indicated that miR-9-3p mimics markedly inhibited the luciferase activity of pmirGLO-MIR22HG-Wt compared to that of pmirGLO-MIR22HG-Mut (Figure 3(e)), suggesting that MIR22HG binds to miR-9-3p. Next, we identified the target mRNAs of miR-9-3p using starBase. After screening (conditions: high stringency of CLIP Data and low stringency of Degradome Data and 2 programs in Program number), AHCYL1, WDR1, and SH2B3 were found. Based on the RT-qPCR results, only SH2B3 was significantly upregulated in OGD/R-treated HL-1 cells (Figure 3(f)). Thus, SH2B3 was selected for further investigations. The binding sequence of miR-9-3p in SH2B3 3′ UTR is shown in Figure 3(g). According to the results of luciferase reporter assay, miR-9-3p overexpression caused a significant reduction of the luciferase activity of SH2B3 3′ UTR wild-type vector (Figure 3(h)), suggesting that miR-9-3p directly targets SH2B3. RT-qPCR and western blotting analyses indicated that either MIR22HG knockdown or miR-9-3p overexpression reduced the expression of SH2B3 at mRNA and protein levels (Figures 3(i) and 3(j)). Collectively, MIR22HG interacts with miR-9-3p to upregulate SH2B3 expression in OGD/R-treated HL-1 cells.

### 3.4. Overexpression of SH2B3 Reverses the Effect of MIR22HG Knockdown in OGD/R-Treated HL-1 Cells

**Figure 4:**
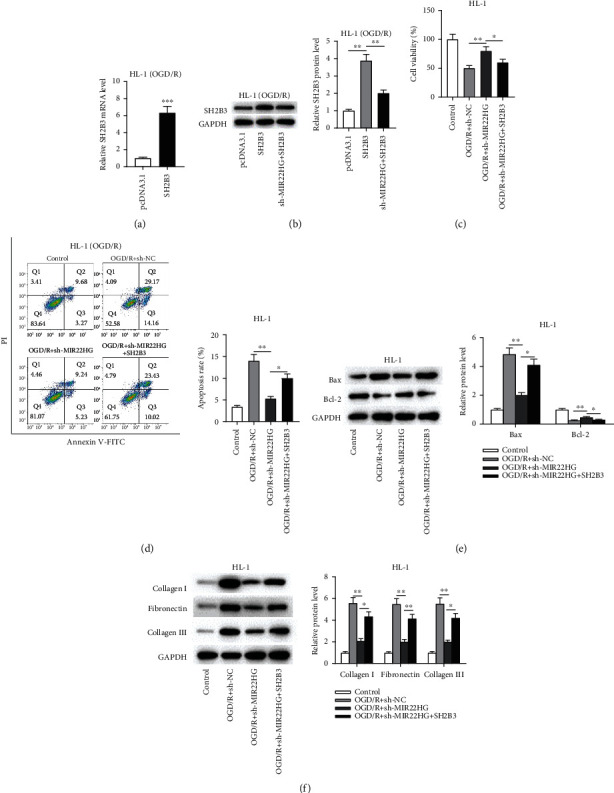
Overexpression of SH2B3 reverses the effect of MIR22HG knockdown in OGD/R-treated HL-1 cells. (a) The overexpression efficiency of pcDNA3.1/SH2B3 was tested by RT-qPCR (*n* = 3). (b) The protein level of SH2B3 in each group was measured by western blotting (*n* = 3). (c) The cell viability in different groups was detected by CCK-8 assay (*n* = 3). (d) Cell apoptosis in different groups was assessed by flow cytometry (*n* = 3). (e and f) The protein levels of Bax, Bcl-2, collagen I, fibronectin, and collagen III in OGD/R-treated HL-1 cells transfected with different vectors were measured by western blotting (*n* = 3). ^∗^*p* < 0.05, ^∗∗^*p* < 0.01, ^∗∗∗^*p* < 0.001.

To validate whether MIR22HG regulates cardiomyocyte phenotypes through the miR-9-3p/SH2B3 axis, rescue assays were designed and carried out. RT-qPCR demonstrated that SH2B3 was markedly overexpressed after transfection of pcDNA3.1/SH2B3 (Figure 4(a)). Compared with the SH2B3 overexpression group, the SH2B3 protein level was decreased after SH2B3 overexpression plus MIR22HG knockdown (Figure 4(b)). The CCK-8 assay revealed that the sh-MIR22HG-mediated promotive effect on cell viability was reversed by SH2B3 overexpression (Figure 4(c)). Additionally, SH2B3 overexpression abrogated the suppressive effect of MIR22HG knockdown on the apoptosis of OGD/R-treated HL-1 cells (Figure 4(d)). Furthermore, the effect of MIR22HG knockdown on the protein levels of cell apoptosis markers and ECM markers in OGD/R-treated HL-1 cells was reversed after overexpressing SH2B3 (Figures 4(e) and 4(f)). All these results confirmed that MIR22HG modulates HL-1 cell apoptosis and ECM production through the miR-9-3p/SH2B3 axis.

### 3.5. MIR22HG Overexpression Reverses the Effect of SH2B3 Knockdown in OGD/R-Treated HL-1 Cells

**Figure 5:**
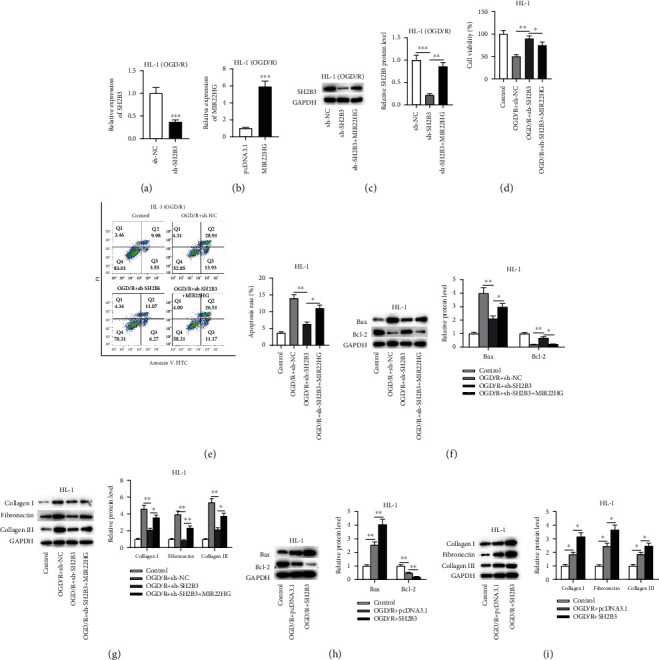
MIR22HG overexpression reverses the effect of SH2B3 knockdown in OGD/R-treated HL-1 cells. (a) The knockdown efficiency of sh-SH2B3 was examined by RT-qPCR (*n* = 3). (b) The overexpression efficiency of pcDNA3.1/MIR22HG was tested by RT-qPCR (*n* = 3). (c) The protein level of SH2B3 in each group was measured by western blotting (*n* = 3). (d) The cell viability in each group was assessed by CCK-8 assay (*n* = 3). (e) The cell apoptosis in each group was determined by flow cytometry analysis (*n* = 3). (f–i) The protein levels of Bax, Bcl-2, collagen I, fibronectin, and collagen III in OGD/R-treated HL-1 cells transfected with different vectors were measured by western blotting analysis (*n* = 3). ^∗^*p* < 0.05, ^∗∗^*p* < 0.01, ^∗∗∗^*p* < 0.001.

Next, the effect of SH2B3 knockdown alone and in combination with MIR22HG overexpression in OGD/R-treated HL-1 cardiomyocytes was investigated. We used RT-qPCR to examine the transfection efficiency of sh-SH2B3 and MIR22HG in HL-1 cells. The results revealed that transfection of sh-SH2B3 effectively reduced the expression of SH2B3 (Figure 5(a)), and transfection of MIR22HG upregulated the expression of MIR22HG in HL-1 cell (Figure 5(b)). Additionally, compared with the SH2B3 knockdown group, the SH2B3 protein level was significantly increased after SH2B3 knockdown plus MIR22HG overexpression treatment (Figure 5(c)). The CCK-8 assay demonstrated that upregulated expression of MIR22HG restored the cell viability inhibited by SH2B3 knockdown (Figure 5(d)). SH2B3 knockdown suppressed the apoptosis of OGD/R-treated HL-1 cells, and this effect was reversed by overexpression of MIR22HG (Figure 5(e)). Furthermore, the effect of SH2B3 knockdown on the protein levels of cell apoptosis markers and ECM markers was attenuated after overexpressing MIR22HG (Figures 5(f) and 5(g)). Overall, MIR22HG overexpression reverses the effect of SH2B3 knockdown in OGD/R-treated HL-1 cells. To verify whether there is a relationship between SH2B3 expression and the increase in cell apoptosis and ECM caused by OGD/R, we examined the effect of SH2B3 overexpression in OGD/R-treated HL-1 cells. The results showed that SH2B3 overexpression significantly increased the protein levels of Bax, collagen I, fibronectin, and collagen III and reduced the protein levels of Bcl-2 (Figures 5(h) and 5(i)), suggesting that SH2B3 overexpression aggravates cell apoptosis and ECM caused by OGD/R.

### 3.6. MIR22HG Knockdown Alleviates Cardiomyocyte Apoptosis in MIRI Mice

**Figure 6:**
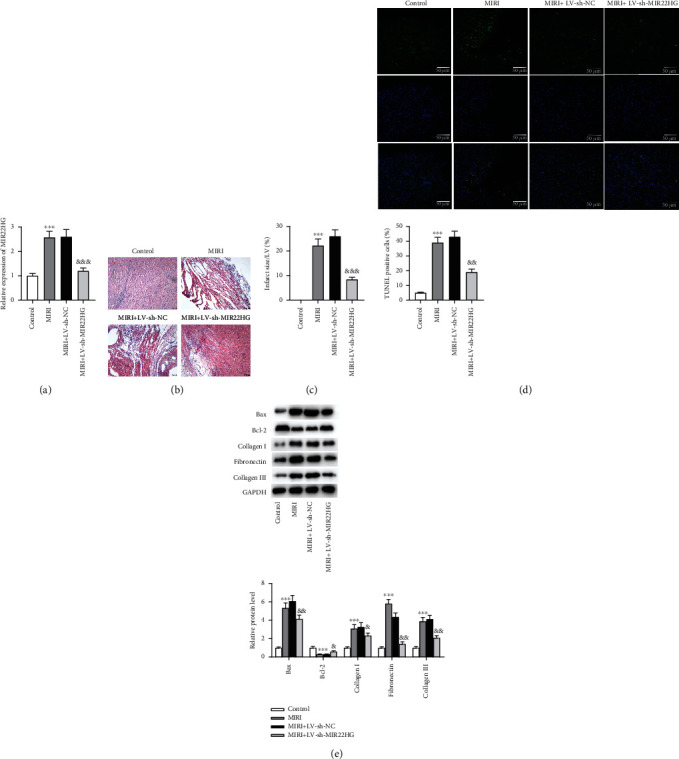
MIR22HG knockdown alleviates cardiomyocyte apoptosis in MIRI mice. (a) RT-qPCR was used to determine the expression of MIR22HG in MIRI mouse models (*n* = 6). (b) HE staining of myocardium tissues (*n* = 6). (c) The analysis of infarct size of MIRI mice (*n* = 6). (d) TUNEL staining was used to assess the apoptosis of cardiomyocytes in each group (*n* = 6). (e) The protein levels of Bax, Bcl-2, collagen I, fibronectin, and collagen III in each group (*n* = 6). *N* = 6 mice for each group. ^∗∗∗^*p* < 0.001 vs. the control group or LV-sh-MIR22HG group; ^&^*p* < 0.05, ^&&^*p* < 0.01, ^&&&^*p* < 0.001 vs. the LV-sh-NC group.

To verify the role of the MIR22HG *in vivo*, animal models of MIRI were established using C57BL/6 mice. We found that the expression of MIR22HG was significantly elevated in infarcted myocardial tissues. Mice were injected with the lentivirus packaging sh-MIR22HG (LV-sh-MIR22HG) or negative control (sh-LV-NC) and then subjected to MIRI. The expression of MIR22HG was downregulated after the injection of LV-sh-MIR22HG (Figure 6(a)). According to the results of HE staining, the number of myocardial cells and blood vessels was reduced in the MIRI group and was significantly restored after the injection of LV-sh-MIR22HG (Figure 6(b)). Additionally, the infarct size was reduced after downregulating MIR22HG (Figure 6(c)), demonstrating the protective effect of MIR22HG knockdown against myocardial injury. The TUNEL staining results showed that MIR22HG knockdown significantly inhibited myocardial apoptosis in MIRI mice (Figure 6(d)). Furthermore, western blotting analysis indicated that MIR22HG knockdown decreased the protein levels of proapoptotic markers and ECM markers in myocardial tissues (Figure 6(e)). Overall, MIR22HG knockdown reduces apoptosis and improves cardiac repair in mice after MIRI.

## 4. Discussion

Almost all cardiovascular surgeries including the surgery of myocardial infarction have been reported to be accompanied by I/R [32]. Unfortunately, I/R is directly related to the occurrence of MIRI [33]. Importantly, cardiomyocyte injury and abnormal deposition of ECM are closely associated with MIRI [34, 35]. In our current study, the HL-1 cells were treated with OGD/R for the establishment of cardiomyocyte injury models *in vitro*, and MIRI animal models *in vivo* were established in male C57BL/6 mice.

Emerging studies have demonstrated that lncRNAs are able to modulate the progression of MIRI by mediating cell proliferation, apoptosis, autophagy, inflammation, and fibrosis [36–38]. For example, inhibition of lncRNA ZFAS1 inhibits cell apoptosis to protect cardiomyocytes against MIRI through the miR-150/CRP axis [39]. Knockdown of lncRNA Mirt1 alleviates MIRI by inhibiting NF-*κ*B activation [40]. Knockdown of lncRNA Gpr19 inhibits cardiomyocyte apoptosis, fibrosis, and oxidative stress in MIRI through the miR-324-5p/Mtfr1 axis [41]. Previous research showed that MIR22HG had a significantly upregulated expression level in AMI patients as compared to healthy samples [21]. Additionally, it was reported that MIR22HG overexpression aggravates hypoxia-induced injury and apoptosis in cardiomyocytes by enhancing NF-*κ*B activation [42]. These studies highlight the research value of MIR22HG in cardiovascular disease. Here, we further explored the expression status, biological function, and molecular mechanism of MIR22HG in MIRI. Consistent with previous studies, our experimental results indicated that MIR22HG showed a significantly high expression level in the *in vitro* and *in vivo* MIRI models. Functionally, MIR22HG knockdown attenuated cell apoptosis and ECM accumulation in OGD/R-treated HL-1 cells, as well as alleviated cardiomyocyte apoptosis and reduced myocardial infarct size in MIRI mice. Therefore, we concluded that MIR22HG knockdown may have a protective role against MIRI.

Mechanistically, a multitude of researches have reported that lncRNAs can form a competitive endogenous RNA (ceRNA) network in which lncRNAs competitively binding to miRNAs to release mRNAs [43] and then regulate gene expression [44]. For example, MALAT1 modulates the apoptosis of cardiomyocytes after hypoxia/reperfusion injury by functioning as a ceRNA through the miR-200a-3p/PDCD4 axis [45]. As for MIR22HG, it has been identified to act as a ceRNA in cancer [31, 46]. In this study, bioinformatics analysis was performed to identify potential miRNAs harboring the binding site for MIR22HG. Then, we validated the binding between miR-9-3p and MIR22HG. Subsequently, SH2B3 was chosen for a target gene of miR-9-3p. We further demonstrated that miR-9-3p directly bound to SH2B3 and negatively regulated the expression of SH2B3. Therefore, MIR22HG acted as a positive regulator of SH2B3 by controlling the availability of miR-9-3p. The adaptor protein Src homology 2-B3 (SH2B3), also known as LNK, is a key regulator in heart diseases including coronary heart disease, peripheral artery disease, and hypertrophy [47, 48]. In detail, SH2B3 aggravates cell fibrogenesis in cardiac hypertrophy [48]. Similarly, in our study, rescue assays indicated that SH2B3 overexpression reversed the inhibitory effect of MIR22HG knockdown on the apoptosis and fibrosis in OGD/R-treated cardiomyocytes. Additionally, the effect of SH2B3 knockdown in cardiomyocytes was reversed by MIR22HG overexpression.

In conclusion, our findings demonstrated that MIR22HG knockdown alleviates myocardial injury *in vitro* and *in vivo* through the miR-9-3p/SH2B3 axis. This evidence highlighted the potential therapeutic benefits by inhibiting MIR22HG during MIRI.

## Data Availability

The datasets used during the current study are available from the corresponding author on reasonable request.
